# Weight-Loss Outcomes: A Systematic Review and Meta-Analysis of Intermittent Energy Restriction Trials Lasting a Minimum of 6 Months

**DOI:** 10.3390/nu8060354

**Published:** 2016-06-08

**Authors:** Michelle Headland, Peter M. Clifton, Sharayah Carter, Jennifer B. Keogh

**Affiliations:** School of Pharmacy and Medical Sciences, University of South Australia, Adelaide 5000, Australia; michelle.headland@mymail.unisa.edu.au (M.H.); sharayah.carter@mymail.unisa.edu.au (S.C.); Jennifer.Keogh@unisa.edu.au (J.B.K.)

**Keywords:** weight loss, diet, intermittent energy restriction

## Abstract

The aim of this systematic review and meta-analysis is to summarise the effects of intermittent energy restriction on weight and biological markers in long term intervention studies of >6 months duration. An electronic search was performed using the MEDLINE, EMBASE and the Cochrane Library databases for intervention trials lasting 6 months or longer investigating the effects of intermittent energy restriction. A total of nine studies were identified as meeting the pre-specified criteria. All studies included an intermittent energy restriction arm, with six being directly compared to continuous energy restriction. A total of 981 subjects were enrolled and randomised, with weight loss observed in all intermittent energy restriction arms regardless of study duration or follow up length. Eight interventions in six trials were used for the meta-analyses, with results indicating neither intermittent or continuous energy restriction being superior with respect to weight loss, 0.084 ± 0.114 (overall mean difference between groups ± standard error; *p* = 0.458). The effects of intermittent energy restriction in the long term remain unclear. The number of long term studies conducted is very limited, and participant numbers typically small (less than 50 completers), indicating the need for larger, long term trials of 12 months or more, to be conducted in order to understand the impact of intermittent energy restriction on weight loss and long term weight management. Blood lipid concentrations, glucose, and insulin were not altered by intermittent energy expenditure in values greater than those seen with continuous energy restriction.

## 1. Introduction

In 2014, the World Health Organisation, classified 39% of the worlds’ adult population as overweight (BMI between 25.0 and 29.9 kg/m^2^) and 13% as obese (BMI ≥ 30.0 kg/m^2^) [1]. Weight loss via dietary restriction is associated with an improvement in biomarkers [2,3]. Cardiovascular disease risk markers including total cholesterol, low-density lipoprotein cholesterol and triglycerides, systolic and diastolic blood pressure, glucose, insulin and C-reactive protein, have been shown to decrease once participants have lost 5% of their body weight [4,5,6,7,8]. Currently, continuous or daily energy restriction is the main form of restriction used by individuals wishing to lose weight via dietary means. This typically involves restricting energy intake by 15%–60% of baseline requirements every day [9]. Whilst continuous energy restriction (CER) has been shown to be an effective weight loss strategy in overweight and obese populations, many individuals find the rigidity of the regime too difficult to maintain [10]. As a result, an alternative dietary regime to the traditional CER, intermittent energy restriction (IER), has gained popularity in the last decade [11]. Various forms of IER are currently being investigated, including alternate-day fasting (ADF). ADF regimes typically involve a “fast day”, where energy intake is either completely withheld or reduced, alternating with “feast” days, on which food is consumed *ad libitum*. A key characteristic of ADF regimes is that frequency of food consumption is changed along with a decrease in overall energy intake [12]. Another form of IER, is the 5 and 2 regime, consisting of five *ad libitum* eating days with two consecutive or non-consecutive “fast” days [13].

A recent review by Seimon *et al.* [14] showed that IER is an effective and comparative (to CER) method for weight loss in the short term, with the most common loss achieved being between 3 and 5 kg after approximately 10 weeks. The ability to maintain weight loss however is a major hurdle in the treatment of obesity, with maximal weight loss commonly achieved at ~6 months after intervention commencement, before a period of weight regain [15,16]. Therefore the purpose of this paper is to provide a systematic review and meta-analysis of studies looking at the long term effects (≥6 month’s duration) of intermittent energy restriction on weight and biological markers in intervention studies and to determine any gaps in the literature which may assist with future study designs.

## 2. Materials and Methods

### 2.1. Data Sources

A systematic search was performed in MEDLINE, EMBASE and the Cochrane Library for original research articles investigating the effects of intermittent energy restriction (IER) published before 30 April 2016. Reference lists of obtained articles were also searched for relevant publications. Studies looking at the intermittent use of very-low-calorie diets were included. No restrictions were placed on publication date, studies were limited to publications written in English, interventions which were a minimum 6 months in duration, and studies in humans. Key search terms were alternate day fast*.tw OR alternat* calori* diet*.tw OR alternate day diet*.tw OR intermittent fast*.tw OR alternate day modified fast*.tw OR intermittent energy fast*.tw OR intermittent energy restrict*.tw OR intermittent energy diet*.tw OR intermittent energy restrict*diet*.tw OR (intermittent adj2 diet*).tw OR intermittent food depriv*.tw OR intermittent calori*restrict*.tw OR (intermittent adj2 restrict*).tw OR VLCD.tw OR very low calorie diet*.tw OR very low energy diet*.tw.

### 2.2. Study Selection

**Figure 1:**
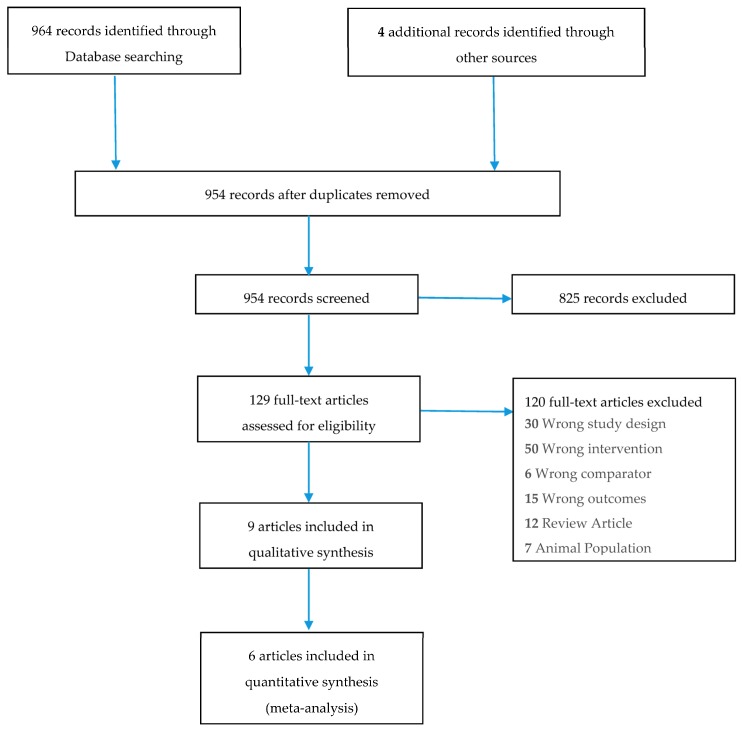
PRISMA Flow Diagram of study selection.

To be included in this review studies were required to meet the following criteria: (1) original article; (2) intervention studies in humans looking at the effect of intermittent energy restriction; (3) weight loss as one of the endpoints; (4) interventions with a minimum 6 months duration. After removal of duplicates, searches identified 964 articles, 825 of these were excluded based on title, leaving 129 possible articles; upon further examination 120 further studies were excluded, leaving nine articles. Figure 1 represents a PRISMA flow diagram of study selection.

### 2.3. Data Analysis

All studies included in the systematic review were deemed to have a low risk of bias (at a study level) as assessed by the “The Cochrane Collaboration’s tool for assessing risk of bias”. To supplement the systematic review a meta-analysis was performed to assess and provide an estimate of the difference in mean weight loss between the CER and IER interventions. Data was analysed using Comprehensive Meta-Analysis (version 2, Biostat, Inglewood, NJ, USA). For each study, the mean weight loss for each group (*i.e.*, IER and CER) was used to calculate the combined overall difference in means between IER and CER.

## 3. Results

### 3.1. Systematic Review

Outlines of the studies focusing on the long term effects of IER, including baseline characteristics, number of study participants and completers, study duration, type of intervention, and weight loss outcomes, are presented in Table 1.

**Figure 2:**
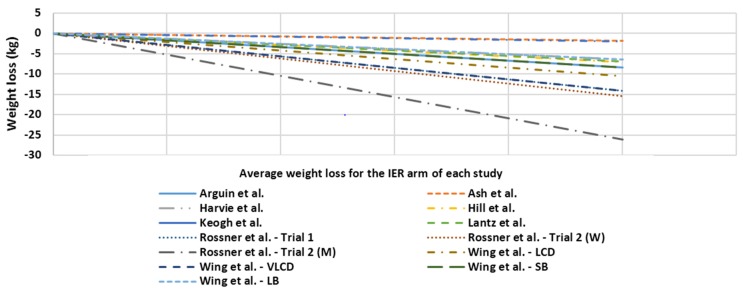
Average weight loss of subjects at the final data collection point for the IER arm of each study.

Of the studies included one of the IER strategies was modified ADF, one was the 5:2 regimen, another two looked at intermittent use of continual energy restriction, one was a week-on, week-off strategy, and the final four had intermittent use of a Very Low Energy Diet (VLED) as the primary IER intervention. A total of 981 subjects were enrolled and randomized with an overall attrition of 39% at study end regardless of duration or follow up length. Weight loss was observed in the IER arms of all studies regardless of study duration. Figure 2 outlines a visual representation of average weight loss at the final data collection point for IER arm for each of the studies.

Of the nine studies included, six compared the intermittent form of energy restriction to continuous energy restriction. Average weight loss achieved by the IER arms in these studies [17,19,20,21,23,25] were comparable to the continuous energy restriction arm, with no significant differences seen between the groups. The intensity of each CER arm varied across each of the studies limiting the opportunity to make direct comparisons between them.

Food was partially supplied [18,23], or supplied in full [22] in three of the studies. Of those three, two of the studies had the primary IER intervention of a VLED program, with the food supplied being liquid meal replacements. Average weight losses experienced in these studies were slightly higher than those seen in studies in which food was self-selected. A possible explanation for this is that the supply of food promoted greater adherence to the intervention. Direct comparisons between those studies which supplied food, or those which didn’t, is difficult due to the differing methodologies used and study duration.

Of those studies that lasted for 12 months of longer (*n* = 6) average weight loss in the IER groups were 8.5 kg [17], 1.8 kg [18], 2.1 kg [21], 7.0 kg [22], 10.5 kg [24], and 6.5 kg or 8.4 kg [25] at the end of their respective follow ups. Whilst all of the studies experienced weight loss at the final follow-up point, this weight loss was lower than that experienced at earlier measurement points in the study. The large variance in results may be partially explained simply due to the length of duration/follow-up, with three studies completing at 12 months [17,21,25], one at 18 months [18], and two at 24 months [22,24]. Other possible explanations include differing drop-out rates, type of volunteer and type of intervention.

#### 3.1.1. Adverse Events

No study reported any serious adverse events related to the dietary intervention. Rossner *et al.* [23] reported that participants found it difficult to embark on the VLED program after experiencing a break. However, participants also stated that an advantage to the intermittent program was that 2 weeks for a VLED program was a suitable duration and one that didn’t require too many lifestyle changes. Harvie *et al.* [19] reported a small number of IER participants (*n* = 4, 8%) experiencing minor adverse physical ailments including feeling cold, constipation, headaches, and lack of energy, whilst a similar number (*n* = 8, 15%) indicated they suffered from minor adverse physiological effects, including a bad temper, lack of concentration and a preoccupation with food.

#### 3.1.2. Study Characteristics and Weight Loss

The difference between groups in weight loss (e.g., CER/control-IER) was not associated with the length of study duration. Further, within each of the IER groups the total length of therapy was not associated with greater weight loss. There were also no associations between BMI or gender and the difference between groups in weight loss in any of the studies. There was no significant difference in weight loss for the studies that received industry support (*n* = 2) compared to those that were not industry funded (*n* = 7). Each of the various forms of intermittent energy restriction employed across the studies were successful in achieving significant (*p* < 0.05) weight loss from baseline. The four studies using intermittent use of VLED achieved mean weight losses of 6.4 kg [19], 14.2 kg [24], 7.0 kg [22], and 14.1 kg in study 1 (females only) and 26.6 kg *vs.* 15.5 kg in study 2 (males *vs.* females respectively) by Rossner *et al.* [23]. The 5:2 intervention saw participants achieve a mean loss of 7.6 kg [20], with that loss similar to those seen in the Ash *et al.* [18] trial looking at the use of ADF of 6.4 kg. Intermittent use of continuous energy restriction (periods of daily moderate energy restriction combined with periods of daily non-restricted eating) saw mean losses of 10.7 kg [17] and 6.5 kg and 8.4 kg [25], with the week-on, week-off strategy achieving a reduction of 2.1 kg in body weight [21].

#### 3.1.3. Drop Outs

From the studies included, the average dropout rate was 31% (ranging from 12% to 65%). Dropouts experienced in each of the studies were similar in the CER and IER groups. Refer to Table 1 (“*N*” Column) for the breakdown of study completers in each arm of the included studies.

### 3.2. Meta-Analysis

**Figure 3:**
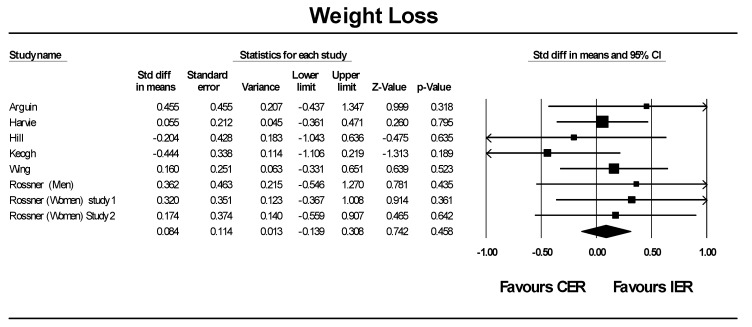
Meta-Analysis: Mean difference in weight loss after IER relative to CER arms within each study.

The variable of interest for the meta-analysis was difference in weight loss between IER and CER/control (Figure 3). From the six papers (eight interventions) included in the analysis the overall effect showed no difference between IER and CER/control in regards to weight loss, 0.084 ± 0.114 (overall mean difference between groups ± standard error; *p* = 0.458). Three studies were not included in the meta-analysis but were retained for qualitative review. First, the study by Lantz *et al.* [22] was excluded from the meta-analysis due to the lack of VLED-free treatment group during the maintenance phase. Overall completers lost 7.0 ± 11.0 kg (6.2% ± 9.5%) in the intermittent group and 9.1 ± 9.7 kg (7.7% ± 8.1%) in the on-demand groups, with no significant difference between groups. Secondly, research by Ash *et al.* [18] was omitted as intervention and control participants were treated as one group during analyses. Lastly, a further study by Wing *et al.* [24] was excluded as the intensity of the IER arm was very different to the intensity of the control arm, despite the length of the two arms being the same. At the end of 50 weeks participants following the VLED program lost more weight than did the LCD subjects (14.2 kg *vs.* 10.5 kg, *p* = 0.057). The complexity of the various diet methodologies, level of energy restriction, and length of follow up across the studies means that the results from this meta-analysis should be considered with caution.

## 4. Discussion

The results of this systematic review of the available literature provides evidence that whilst IER is not superior to CER, it is as effective for weight loss. The effect of the IER regimens on weight included in this reviews are varied, most likely due to the diverse range of dietary methods used. Currently there is insufficient data to support the notion that IER (in any form) can affect CVD risk markers (*i.e.*, blood pressure and blood lipid levels) or insulin and glucose to a greater extent than that seen with CER. Moreover, changes in HbA_1_c or insulin sensitivity levels beyond weight loss have not been established. Further investigations into this are needed.

With weight loss appearing to plateau at 6 months, it is important that weight loss interventions not only focus on weight loss, but on weight maintenance [26]. Weight regain is likely to occur if a dietary intervention is stopped entirely, therefore an emphasis on a continuing lower-energy diet in combination with regular exercise is important. A systematic review (80 studies, *N* = 26,445; 18,199 completers (69%)) and meta-analysis (47 studies, *N* = 5409 completers) by Franz *et al.* [27] reported overweight or obese participants (aged 18 years or older) undergoing various weight-loss interventions-diet alone, diet and exercise, and meal replacements, experiencing a mean weight loss of 5 to 8.5 kg (5% to 9%) during the first 6 months of the interventions, with weight plateau at 6 months. In the interventions lasting 48 months (a mean of 3 to 6 kg (3% to 6%) weight loss was maintained with no groups experiencing weight regain back to baseline levels. Similarly Curioni and colleagues [28] performed a review of clinical trials looking at long-term weight loss after diet and exercise and reported a mean weight loss of 6.7 kg after 1 year in individuals undergoing combined exercise and diet therapy.

The American Dietetic Association’s Adult Weight Maintenance Evidence-Based Nutrition Practice Guidelines recommend an optimal weight loss target of 0.5 to 1 kg per week for the first 6 months and to achieve an initial weight-loss goal of up to 10% from baseline [29]. Weight loss in several of the included studies is less than these guidelines, however the level of supervision or contact with the research group and physical activity guidelines set may play a role as this varied considerably in the studies. The concept of extended care comes from the continual care model [30] through which individuals receive continued contact with therapists following an initial period of weight loss. Common forms include in-person sessions, either individual or groups, or telephone contact, and are designed to reinforce strategies put in place during the intervention and provide continuing motivation. This motivation may prove to be especially important for maintaining behavioural changes during periods of weight loss plateau or when individuals are moving from weight loss to weight maintenance. A recent systematic review and meta-analysis of randomized controlled trials investigating the effect of extended care reported that the average effect size of extended care on weight loss maintenance using Hedge’s *g* = 0.385 (*p* < 0.0001), leading to the maintenance of an additional 3.2 kg weight loss over 17.6 months post intervention compared to controls [31] highlighting that contact with the research group during IER interventions could play a significant role in overall results achieved. As only 11 studies were included in the review however, the impact of the type of contact (e.g., phone or in person) or frequency of contact (weekly or monthly) remains unknown. Pooled results from eight studies in a review by Johns and colleagues [32], looking at the effect of combined behavioural weight management programs (BWMPs), which provide diet interventions combined with physical activity, showed that whilst there is no difference in weight loss at 3 to 6 months compared to diet only arms, BWMPs produce a significantly greater weight loss at 12 months (−1.72 kg).

All the studies included an obese but healthy population creating a potential gap in the wider applicability of the results. Future studies should research IER in other sub groups of the population, e.g., people with diabetes or heart disease. Intermittent VLED interventions included in this review have shown a positive effect on weight loss in well controlled studies. However the few long-term studies available means it is difficult to determine the long-term applicability of this type of regime. In interpreting results it is important to note differences in diet methodology as well as study design between studies in assessing the impact of IER, which may limit the overall outcomes.

There are several limitations to this systematic review and meta-analysis that need to be recognised. Data extracted is restricted to data reported in primary studies and it is difficult to account for any potential bias for publishing studies that favour successful interventions or the potential for enrolling only participants that are the most likely to succeed. Assuming however that the inclination to enroll subjects likely to be successful is distributed evenly across all intervention groups, trial comparisons are still appropriate. For most of the studies included in this review the number of participants screened prior to study commencement has not been reported. If these numbers had been reported it is likely that the numbers and therefore overall percentages of individuals who complete the study would be significantly lower. Some of the included studies may not have been adequately powered to detect differences in body weight between CER and IER groups, with only three trials reporting power calculations [18,19,21]. This combined with the small number of studies that fitted the criteria limits the applicability of the findings. Additionally, difference in IER form, overall study design, and participant numbers makes it difficult to fully identify which form of IER is most effective for weight loss. Whilst a meta-analysis was performed comparing IER to CER, all studies were highly variable in terms of prescribed energy restriction, diet composition, and timing of the intermittent periods. Despites these limitations, this review and meta-analysis provides valuable information into the growing field of IER, presenting a summary of studies that have included both a weight loss and weight maintenance phase.

## 5. Conclusions

This review confirms that in the little long term evidence available, IER achieves weight loss but there was no evidence that it provided superior management in comparison to CER. Furthermore dropout rates were similar in the IER and CER arms of the included studies, suggesting that long term adherence to IER may be similar to CER and therefore present a successful alternative for individuals who find CER too restrictive in dietary choices during weight reduction. Larger, longer-term trials of 12 months or more are needed to fully investigate the effects of IER on weight loss, weight management, and diet sustainability. Studies of intermittent VLED use have shown positive results but further longer-term trials are required to fully appreciate any lasting benefits they may produce.

## Figures and Tables

**Table 1:** Study Outlines.

Reference	Study Design	Study Participants	*N*		Prescribed Regimen	Outcome Measures	Intervention Adherence	Effects of Intervention	Weight Change
			Start	End					
Arguin *et al.* [17]	Randomized, controlled, parallel study of 5 weeks intervention, 5 weeks stabilization phase, and 1 year follow up.	F Age: Mean 60.5 years BMI: Obese	22 IER: 12 CER: 10	20 IER: 11 CER: 9	*Intervention* Intermittent Diet (IER)—2 cycles of 5 weeks of weight maintenance plus 5 weeks of moderate CER OR Continuous energy restriction (CER)—15 weeks of moderate CER	BW, BC, WC, RMB, lipid profile, glucose	Not reported.	↓^†^ seen in TC and triglycerides in both groups after initial 5 weeks intervention.	*At the 1 year follow up* **(mean ± SD)**: ↓ 8.5 ± 4.2 kg (IER) ↓ 7.1 ± 4.7 kg (CER) *p* = 0.73 between groups.
Ash *et al.* [18]	Randomised, controlled, parallel-arm study of 3, 12 weeks interventions and 18 months follow up	M Age: >70 years BMI: 25–40 kg/m^2^ T2DM	51	27 *	*Intervention* 4200 kJ/day for 4 consecutive days, 3 days *ad libitum* eating (Food provided on fasting days) OR PPM; Removal of food preparation cues (All food provided) *Control group* SSM; Prepare and select own foods (Food not provided).	BW, BC, WC, HbA1C, triglycerides	Not reported.	Mean ↓ in energy intake (2369 ± 2793 kJ/day, *p* < 0.001)), HbA1C (1.0% ± 1.4%, *p* < 0.001), WC (8.1 ± 46 cm, *p* < 0.001), % body fat (1.9% ± 1.5%, *p* < 0.001), and triglyceride levels (0.3 ± 0.6 mmol/L, *p* = 0.02) post 12 weeks intervention from baseline. No improvements were maintained at the 18 months follow up visit.	*Mean Outcome Measures:* Baseline: 98.5 ± 12.3 kg* 12 weeks: 92.1 ± 11.4 kg* (*p* < 0.001 from baseline) 18 months: 96.7 ± 12.1 kg* (NS from baseline)
Harvie *et al.* [19]	Randomised, controlled, parallel study of a 6 months intervention	F Age: 30–45 years BMI: 24–40 kg/m^2^	107 IER: 53 CER: 54	89 IER: 42 CER: 47	*Intervention* 75% ER (~2710 kJ/day) for 2 non consecutive days/week and CER on the other 5 days-IER (Food not provided) OR 25% ER (~6276 kJ/day) for 7 days/week-CER (Food not provided)	BW, insulin sensitivity and metabolic disease risk markers	*IER* 70% completed 2 VLED days/week at 1 month, 56% at 3 months and 64% at 6 months. *CER* 71%, 61% and 55% at 1, 3 and 6 months respectively.	Comparable ↓ noted for leptin, CRP, TC, LDL-C, triglycerides, and BP compared to baseline values for each group. IER intervention resulted in greater reductions (*p* < 0.05) in fasting insulin (2.1 µU·mL^−1^) and insulin resistance (0.4 µU·mmol^−1^·L^−1^) compared to CER (1.1 µUmL^−1^ and 0.3 µU·mmol^−1^·L^−1^) after 6 months. Tests were performed on a non-fasting day a minimum 5 days post weekly VLED treatment. *Acute Response of serum markers* A subset (15 IER and 9 CER) provided fasting samples the morning after a 2-day VLED which showed acute reductions for the IER group in fasting insulin (−23%), HOMAR (−29%) and TC (−18%). No changes reported in CER group.	*At 6 months* **(mean & range)**: ↓ 6.4 (↓ 7.9 to ↓4.8 ) kg (IER) ↓ 5.6 (↓6.9 to ↓4.4) kg (CER) *p* = 0.26
Hill *et al.* [20]	Randomised four-arm parallel study of 12 weeks with follow up at 26 weeks	F Age: ≥18 years Weight: 130%–160% of ideal body weight	40 IER: 20 CER: 20	32 IER: 16 CER: 16	*Intervention* Severe ER (2512 kJ/day) on fast days, moderate CER (7536 kJ/day) on alternating days–IER IER plus moderate aerobic training on 5 days/week—IER + EX OR Moderate CER (5024 kJ/day)—CER CER plus moderate aerobic training on 5 days/week—CER + EX	BW, BC, REE, TC, Triglycerides, glucose, insulin	95% of participants who completed the study.	FM ↓ during the study with no difference between diets (6.1 ± 0.6 *vs.* 6.0 ± 0.8 kg for constant and alternating dies respectively. REE ↓ by 5% in the group as a whole (*p* < 0.05), with no diet or exercise effects TC ↓ in the group as a whole (5.30 ± 0.18 to 4.73 ± 0.18 mmol/L, *p* < 0.05). Subjects in the IER conditions showed greater reductions than did the subjects following CER conditions (5.46 ± 0.26 to 4.68 ± 0.23 mmol/L *vs.* 5.09 ± 0.23 to 4.81 ± 0.26 mmol/L, *p* < 0.05) Triglycerides ↓ with weight reduction (1.21 ± 0.11 to 1.02 ± 0.11 mmol/L, *p* < 0.05) with no diet or exercise effect. Fasting glucose ↑ without any change in fasting insulin, with no diet or exercise effect.	*Following the 12-week intervention:* Total weight loss during 12 weeks was 7.6 kg (NS between diets). *At the six-month follow up* **(mean ± SEM):** ↓ 9.5 ± 2.9 kg (CER) ↓ 7.2 ± 2.7 (IER) NS difference between groups
Keogh *et al.* [21]	Parallel, randomized control trial, of 8 weeks with a 12-month follow-up.	F Age: ≥18 years BMI: >27 kg/m^2^	75 IER: 39 CER: 36	36 IER: 19 CER: 17	*Intervention* Intermittent Energy Restriction (IER) 5500 kJ/day for 1 week followed by 1 week of usual dietary habits OR Continuous Energy Restriction (CER) 5500 kJ/day for the duration of the study	BW, BC, diet quality scores	24 women (12 from each treatment group) did not adhere to the diet for the 44 weeks between 8 weeks and follow-up. 11 reported continuing their allocated diets for the duration of the study (4 CER, 7 IER).	↓ in waist and hip circumference over time (*p* < 0.01) in both groups, no difference between groups. ↑ in Healthy eating index at 12 months in the CER compared to IER (8.4 ± 9.1 *vs.* −0.3 ± 8.4, *p* = 0.006).	In completers only: *After 8 Weeks* **(mean ± SD)**: ↓ 3.2 ± 2.1 kg (CER) ↓ 2.0 ± 1.9 kg (IER) *p* = 0.06 between groups. *12 months follow-up* **(mean ± SD)**: ↓ 4.2 ± 5.6 kg (CER) ↓ 2.1 ± 3.8 kg (IER) *p* = 0.19 between groups.
Lantz *et al.* [22]	Randomised, parallel study of 2 years trial.	MF Age: 18–60 years BMI: >30 kg/m^2^	334 IER: 161 On-Demand: 173	117 IER: 57 On-Demand: 60	VLED (1890 kJ/day) for 16 weeks, VLED for 2 weeks every third month (Intermittent). OR VLED for 16 weeks, VLED on-demand when body weight increased above desired cut-off (On demand).	BW, FFM, BP, glucose, insulin, TC, HDL-C, LDL-C, triglycerides	Not reported.	No significant differences between the groups at baseline or over time. Completers were pooled from both groups to show changes of the following variables: ↓ in systolic (−7 mmHg) and diastolic (−3 mmHG) BP after 1 year ^†^ but not after 2 years. TC ↓^†^ after 24 weeks (−0.2 mmol L^−1^) but not at 1 or 2 years. HDL-C and LDL-C ↓ by 0.2 mmol L^−1^ and −0.2 mmol L^−1^ respectively after 100 weeks ^†^. ↓ in glucose, insulin and relative insulin by −0.4 mmol L^−1^, −7.6 mU L^−1^, and −2.1 respectively after 48 weeks ^†^. With only serum insulin (−4.9 mU L^−1^) and relative insulin resistance (−1.1) remaining improved after 100 weeks ^†^	*At the end of 2 years* **(mean ± SD)**: ↓ 7.0 ± 11.0 kg (Intermittent) ↓ 9.1 ± 9.7 kg (On-demand) NS between groups
Rossner *et al.* [23]	Randomised parallel, controlled trial of 18 weeks with follow up at 14 and 26 weeks	MF Age: 21–60 years BMI: >30 kg/m^2^	101 IER: 20 (Trial 1) 29 (Trial 2) Control: 20 (Trial 1) 32 (Trial 2)	81 IER: 17 (Trial 1) 22 (Trial 2) Control: 16 (Trial 1) 26 (Trial 2)	TRIAL 1—Women only *Intervention* Three periods of 2 weeks with VLED (1764 kJ/day) separated by 4 weeks of moderate CER (6592 kJ/day) *Control* Six weeks continuous treatment with VLED (1764 kJ/day) TRIAL 2—Men and Women *Intervention* Three periods of 2 weeks with VLED (2226 kJ/day) separated by 4 weeks of moderate CER (6592 kJ/day) *Control* Six weeks continuous treatment with VLED (2226 kJ/day)	BW	Measured via urinary ketone bodies, but results not reported.	-	*Mean outcome measures:* **(mean weight ± SD)** TRIAL 1—Women only *Intervention* Baseline; 106.2 ± 14.2 kg At 26 weeks; 92.1 ± 14.6 kg *Control* Baseline; 105.6 ± 10.5 kg At 26 weeks; 95.5 ± 11.1 kg TRIAL 2 *Intervention* Baseline; Men; 135.0 ± 21.6 kg Women; 114.5 ± 13.0 kg At 26 weeks: Men; 108.8 ± 24.4 kg Women; 99.0 ± 16.8 kg *Control* Baseline: Men; 127.4 ± 9.6 kg Women; 107.7 ± 17.6 kg At 26 weeks: Men; 107.7 ± 12.9 kg Women; 94.9 ± 12.6 kg NS difference between groups.
Wing *et al.* [24]	Randomised, two—arm parallel study of 50 weeks + 2 years follow up.	MF BMI: >30 kg/m^2^	93 IER: 45 CER: 48	79 IER: 38 CER: 41	*Intervention* LED (4200–5040 kJ/day) for the whole 50 weeks (LED) OR 2 × 12 weeks periods of VLED (1680–2100 kJ/day) alternating with the balanced LED (VLED).	BW, glucose, BP, lipids	Not reported.	↓ in cholesterol for both groups from baseline after 1 year ^†^ with greater difference seen in the LCD group (*p* = 0.058) Significant and comparable ↑in HDL-C and ↓ in triglycerides after 1 year ^†^ ↓ in HBA_1_C levels of 10.6% to 8.3% and 10.2% and 8.8% for the VLED and LED groups respectively (*p* <0.001). No difference between the groups. ↓ in fasting glucose and insulin levels from baseline after 1 year ^†^ for both groups. Changes were comparable for the two treatments At the 2 years follow up levels of fasting glucose, HBA_1_C and insulin levels were comparable between the two groups.	At 50 weeks follow up **(mean ± SD)**: ↓ 14.2 ± 10.3 kg (VLED) ↓ 10.5 ± 11.6 kg (LED) *p* = 0.057
Wing *et al.* [25]	Randomised 3-arm parallel study of 20 weeks IER and 14 weeks CER, with follow-up at 20 & 48 weeks.	MF BMI: >30 kg/m^2^	142 IER: 47 (LB) ^∏^ 47 (SB) Control: 48	96 IER: 32 (LB) 33 (SB) Control: 31	*Intervention* 7 weeks of moderate CER (4200–6300 kJ/day), 6 weeks break, 7 weeks of moderate CER—Long Break IER (LB) 3 cycles of 3 weeks of moderate CER (4200–6300 kJ/day), 2 weeks break, 5 weeks of moderate CER—Short Break IER (SB) *Control* 14 weeks moderate CER (4200–6300 kJ/day) plus restriction of 13 specified high-fat foods		68% of Long Break IER, 70% Short Break IER, & 64% CER		Posttreatment (5 months) **(mean ± SD)**: ↓ 8.2 ± 3.7 kg (Control) ↓ 7.0 ± 5.0 kg (LB) ↓ 8.2 ± 6.3 kg (SB) NS significant difference between groups At 48 weeks follow up **(mean ± SD)**: ↓ 7.3 ± 5.1 kg (Control) ↓ 6.5 ± 5.9 kg (LB) ↓ 8.4 ± 8.2 kg (SB) NS difference between groups.

* Please note: all participants analysed as a whole group. PPM, removal of food preparation cues; SSM, usual dietetic intervention. ^∏^ LB, long break; SB, short break. ^†^ *p* < 0.05.

## Abbreviations

The following abbreviations are used in this manuscript:

BC	Body composition
BMI	Body mass index
BP	Blood pressure
BW	Body weight
BWMP	Behavioural weight management programs
CER	Continuous energy restriction
CRP	C-reactive protein
FFM	Fat free mass
FM	Fat mass
HbA1C	Glycated haemoglobin
HDL-C	High density lipoprotein cholesterol
HOMAR	Homeostatic model assessment of insulin resistance
IER	Intermittent energy restriction
LDL-C	Low density lipoprotein cholesterol
LED	Low energy diet
REE	Resting energy expenditure
SD	Standard deviation
TC	Total cholesterol
VLED	Very low energy diet
WC	Waist circumference
